# Conversion to Sirolimus Therapy in Kidney Transplant Recipients with New Onset Diabetes Mellitus after Transplantation

**DOI:** 10.1155/2013/496974

**Published:** 2013-05-20

**Authors:** Massimiliano Veroux, Tiziano Tallarita, Daniela Corona, Nunziata Sinagra, Alessia Giaquinta, Domenico Zerbo, Carmela Guerrieri, Antonino D'Assoro, Sebastiano Cimino, Pierfrancesco Veroux

**Affiliations:** ^1^Vascular Surgery and Organ Transplant Unit, Department of Surgery Transplantation and Advanced Technologies, University Hospital of Catania, 95123 Catania, Italy; ^2^Medical Oncology, Department of Biochemistry and Molecular Biology, Mayo Clinic, Rochester, MN, USA

## Abstract

New-onset diabetes mellitus after transplantation (NODAT) may complicate 2–50% of kidney transplantation, and it is associated with reduced graft and patient survivals. In this retrospective study, we applied a conversion protocol to sirolimus in a cohort of kidney transplant recipients with NODAT. Among 344 kidney transplant recipients, 29 patients developed a NODAT (6.6%) and continued with a reduced dose of calcineurin inhibitors (CNI) (8 patients, Group A) or were converted to sirolimus (SIR) (21 patients, Group B). NODAT resolved in 37.5% and in 80% patients in Group A and Group B, respectively. In Group A, patient and graft survivals were 100% and 75%, respectively, not significantly different from Group B (83.4% and 68%, resp., *P* = 0.847). Graft function improved after conversion to sirolimus therapy: serum creatinine was 1.8 ± 0.7 mg/dL at the time of conversion and 1.6 ± 0.4 mg/dL five years after conversion to sirolimus therapy (*P* < 0.05), while in the group of patients remaining with a reduced dose of CNI, serum creatinine was 1.7 ± 0.6 mg/dL at the time of conversion and 1.65 ± 0.6 mg/dL at five-year followup (*P* = 0.732). This study demonstrated that the conversion from CNI to SIR in patients could improve significantly the metabolic parameters of patients with NODAT, without increasing the risk of acute graft rejection.

## 1. Introduction

Kidney transplantation (KT) is the best available therapy for end-stage renal disease. Recent improvements after kidney transplantation are due to the introduction of more effective immunosuppressive agents and improved surgical techniques [1–5]. However, a similar improvement in long-term graft survival has not been observed and the complications related to the posttransplant immunosuppressive therapy remain common [1, 6, 7].

New-onset diabetes mellitus after transplantation (NODAT) is a well-recognized complication associated with reduction in both graft and patient survivals [1, 8, 9]. Data from the US Renal Data System (USRDS) indicate that 40% of KTs will have developed NODAT by their third year aftertransplantation [10]. New-onset diabetes mellitus is a major risk factor for cardiovascular disease [11–13] and mortality [1, 8, 12–15] and is also associated with reduced kidney graft survival [16, 17], infections [1, 8, 18], and increased health care costs [19]. A number of risk factors have been identified: they include obesity, age, ethnicity, family history, donor source, race, polycystic kidney disease, hepatis C seropositivity,* TCF7L2* polymorphism, the Fok1 VDR polymorphism [1, 7, 20–28], and the type of immunosuppressive agents used to prevent and treat acute rejection [1, 6, 10, 20, 24–28].

The extent to which the immunosuppressive agents may induce diabetes is extremely variable, so that the choice of immunosuppressive therapy may have a strong impact on recipient's risk to develop a NODAT. In the metanalysis performed by Montori et al. [29], the type of immunosuppressive regimen used determined 74% of the variability in incidence of NODAT between different studies, with high-dose steroids being associated with the highest incidence. 

The use of calcineurin inhibitors (CNI) led to an increased risk for diabetes after transplantation; the risk being higher for tacrolimus than cyclosporine [1, 3, 7, 20, 21]. The recent development of immunosuppressive protocols with the intent to minimize the use of CNI and steroids had stimulated the extensive use of potent nonnephrotoxic immunosuppressant, such as mycophenolate mofetil (MMF) and sirolimus [30, 31].

The evidence that diabetes was not increased when sirolimus was added to cyclosporine and steroids [32], the similar incidence of NODAT reported for sirolimus when compared to cyclosporine [33], and the evidence that rapamycin may prevent the development of NODAT after kidney transplantation [9] stimulated a protocol of conversion from CNI- to sirolimus-based immunosuppression in kidney transplant recipients who developed NODAT. We present the results of such study of conversion, by evaluating the rate of remission and the impact of sirolimus on the management of NODAT. 

## 2. Patients and Methods

This was a retrospective study of all consecutive patients with end-stage renal disease, who received kidney transplantation at the Organ Transplant Unit of the University Hospital of Catania between January 2001 and April 2008. A total of 344 kidney transplantations (259 from deceased donor and 75 from living donor) were reviewed. Patients with a diagnosis of diabetes mellitus as a cause of end-stage renal disease were not included in this study.

Preoperative assessment in each patient included age, sex, year of transplantation, number of donor HLA A, B and DR mismatch, time of initiation and type of dialysis, history of myocardial infarction, stroke, and extensive cardiovascular assessment (thallium scintigraphy and/or coronary angiography).

All patients on the waiting list underwent on a three-month basis a fasting plasma glucose (FPG) measurement: patients with normal FPG levels (<100 mg/dL) were included in the active list for transplantation; those patients with FPG values >100 mg/dL underwent an extensive metabolic evaluation to rule out diabetes mellitus, including serial fasting glycaemia levels, C-peptide, oral glucose tolerance test, and HbA1C levels [34–36].

In the posttransplant immunosuppression protocol, all patients received a dose of 750 mg of prednisolone (STER) at the time of transplant and then a dose of 1 mg/Kg per day, which was slowly tapered to a maintenance dose of 5 mg/day by the end of the sixth month. Mycophenolate mofetil (MMF) was given at a dose of 1 to 2 g/day. For patients receiving Tacrolimus-based immunosuppression, tacrolimus was initiated at 0.1 mg/Kg/die and doses were adjusted to keep levels between 10 and 12 ng/mL in the first month post-transplant and between 8 and 10 ng/mL thereafter. For recipients receiving cyclosporine-based immunosuppression, cyclosporine (CyA) was started 2 days after operation at 5 mg/kg/die and doses were adjusted to keep levels at 200–220 ng/mL for the first three months after the transplant, followed by doses of 150 to 200 ng/mL between 3 and 6 months after the transplant, and more than 140 ng/mL thereafter. Sirolimus (SIR) was initiated at 5-6 mg, beginning within day 5 post-transplant, and doses were adjusted to keep levels between 8 and 12 ng/mL.

In all patients in whom an acute rejection was suspected, a graft biopsy was obtained and the rejection scored according to the Banff classification. Rejection therapy consisted in steroid pulses of 500 mg of methylprednisolone for three days.

Fasting blood glucose levels were collected daily after transplantation, and with each outpatient biochemical assessment. The diagnosis of new-onset diabetes mellitus was based on a fasting plasma glucose levels ≥126 mg/dL or a non-fasting plasma glucose level of >200 mg/dL in at least 2 repeated measurements [34–36].

Patients with a diagnosis of NODAT were divided into two groups: first eight patients (group A) underwent a “standard” therapy with reduction of CNI's levels by 20% to achieve trough levels of 5–7 ng/mL in tacrolimus-based immunosuppression, and 130–150 mg/dL in cyclosporine-based immunosuppression; in all other patients (group B) a conversion protocol was applied: the CNI (Tacrolimus or cyclosporine) was abrupt converted to SIR, and all recipients received a single oral loading dose of SIR of 5 mg. Whole-blood SIR trough concentration first was measured on a fifth day after the conversion, and the SIR daily dose was modified to achieve target trough levels of 7 to 10 ng/mL.

All patients with a diagnosis of NODAT were admitted to the transplant unit. Glucose levels were assessed three times a day by finger-prick blood glucose measurement, while fasting plasma glucose levels were assessed on a daily basis until discharge.

Insulin was administered endovenous and then intra muscular at a dose able to achieve a fasting blood glucose levels below 110 mg/dL. Resolution of NODAT was defined as cessation of insulin requirement and a fasting glucose level <110 mg/dL. 

### 2.1. Statistical Analysis

End-points included freedom from NODAT, patient and graft survival between CNI- and m-TOR inhibitor- based immunosuppression. Follow-up was extended to five years after transplantation. Results were reported as percent or Odds ratio (OR) with 95% confidence interval (95% CI). The Pearson *χ* ^2^ or Fisher exact test was used for analysis of categorical variables. Differences between means were tested with two-sided *t* test, the Wilcoxon rank sum test, or the Mann-Whitney test. A value of *P* < 0.05 was used to determine statistical significance. Independent risk factors for NODAT were analysed by univariate and multivariate logistic regression analysis.

## 3. Results

New-onset diabetes mellitus was diagnosed in 29 (6.6%) patients (Table 1). The incidence of NODAT was similar in patients treated with tacrolimus (22 patients, 7.7%) and cyclosporine (7 patients, 7%), while no patient on *de novo* sirolimus therapy developed post-transplant diabetes. Mean time of onset of NODAT was 9 ± 4.2 months after kidney transplantation. Although not statistically significant, NODAT was more common in male and in patients with polycystic kidney disease.

Most of the patients presented with a severe hyperglycaemia, which required an intensive care monitoring and an aggressive insulin therapy to normalize the blood glucose levels. Mean fasting glucose level at admission was 209 ± 45 mg/dL. 

Rescue treatment was based on lowering CNI dosage by 20% in 8 (28%) patients and abrupt switch to sirolimus in 21 (72%). 

There were no recorded acute rejection episodes during the followup. 

New-onset diabetes mellitus resolved in 3 patients in group A (37.5%) and in 17 patients in group B (80%) after a mean time of 13 ± 4.5 months from its onset.

Five-year patient and graft survivals of the entire study group were 89.7% and 79.4%, respectively. There were two patients who died with a functioning graft (death-censored graft survival 85.2%), for an intestinal infarction and acute myocardial infarction. One more patient died due to acute hepatic failure three months after graft failure. 

Unexpectedly, overall five-year graft and patient survival in patients who did not develop NODAT were similar to NODAT+ patients: 94% and 79.1%, respectively (*P* = 0.623). This may be partially explained by the small sample size of NODAT+ group and, probably, by the high rate of complete resolution of NODAT in our transplant population.

At five-year followup, group A patient and graft survivals were 100% and 75%, respectively, not significantly different from group B patient (83.4% and 68%, respectively, *P* = 0.847). 

**Figure 1:**
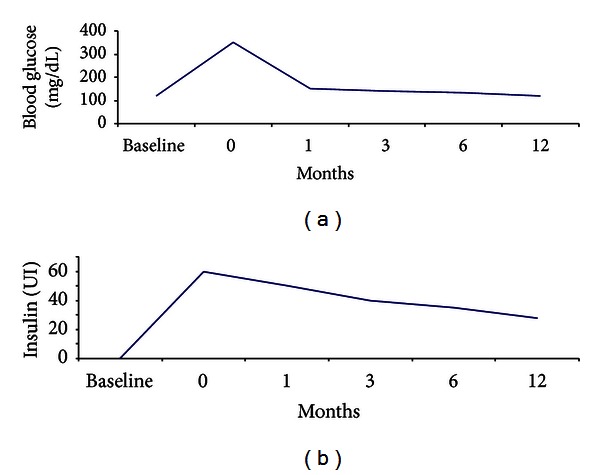
Fast plasma glucose levels and insulin requirement in patients with new-onset diabetes mellitus, measured after and before conversion to sirolimus. The time of conversion was indicated as time 0. Data are expressed as mean values.

In group A fasting plasma glucose levels decreased to 141 ± 36 mg/dL in the meantime of 3 ± 5 months, while in group B, fasting plasma glucose levels decreased to 135 ± 17 mg/dL in a meantime of 4 ± 3 months (*P* < 0.05) (Figure 1). 

Interestingly, graft functionality improved after conversion to sirolimus therapy: serum creatinine was 1.8 ± 0.7 mg/dL at the time of conversion and 1.6 ± 0.4 mg/dL five years after conversion to sirolimus therapy (*P* < 0.05); in the group of patients remaining with a reduced dose of CNI, graft functionality did not change significantly over time: serum creatinine was 1.7 ± 0.6 mg/dL at the time of conversion and 1.65 ± 0.6 mg/dL at five-year followup (*P* = 0.732). 

## 4. Discussion

This is the first study investigating the role of a conversion protocol to sirolimus in the management of new-onset diabetes mellitus after kidney transplantation. 

New-onset diabetes mellitus is a form of type 2 diabetes, which is thought to develop in response to a relative insulin deficiency resulting from increased insulin resistance or impaired insulin production [35, 36]. Kidney transplant recipients are particularly at risk to develop such a common complication, as a consequence of factors additional to those typical of general population, including the use of immunosuppressive agents [37].

The incidence of new-onset diabetes mellitus is extremely variable between the studies: a recent meta-analysis of observational studies reported that the incidence of NODAT in the first year after transplantation varied from 2% to 50% [38], with the type of immunosuppression having the strongest impact on the incidence of NODAT. Corticosteroids are associated with the highest risk of NODAT after transplantation, and their effect is dose dependent [3, 39], by stimulating insulin resistance [40]. There have been several reports of reduced incidence of NODAT with early withdrawal of steroids and using dual therapy with MMF and tacrolimus [41]. In a study comparing a corticosteroids-free regimen of tacrolimus, MMF and daclizumab induction therapy with tacrolimus, and MMF and corticosteroids, Rostaing et al. [42] did not found difference in acute rejection episodes between the two groups but the incidence of NODAT was 5.4% in the steroid-containing regimen and 0.4% with the steroid-free regimen. However, other studies with steroid-free regimens with tacrolimus did not confirm this observation [3, 43].

More recently, Luan et al. [44] studying the relationship between steroid-free immunosuppression in a cohort of 25,837 previously nondiabetic kidney transplant recipients found that the cumulative incidence of NODAT within 3 years of transplant was 17.7% with maintenance steroids and 12.3% without (*P* < 0.001). Patients discharged with steroids had 42% greater odds of developing NODAT compared with those without steroids, and the risk was higher in patients treated with tacrolimus. 

Both cyclosporine and tacrolimus are associated with increased NODAT risk. Most of the studies report a greater risk for tacrolimus than cyclosporine [1, 3, 7, 20, 21]: however, a series comparing tacrolimus- with cyclosporine-treated kidney transplant recipients demonstrated that the only significant differences between the two groups may be seen within 3 months after transplant, and no other significant differences between tacrolimus- and cyclosporine-treated patients for any of the glucose metabolism parameters (blood glucose levels, C-peptide secretion) appeared during the rest of the 3-year followup [45]; moreover, despite the association between tacrolimus and NODAT and the association between NODAT and reduced graft survival, tacrolimus is nevertheless associated with improved graft survival [1]. Therefore, it is unlikely that renal transplant recipients who experience NODAT three months aftertransplant, will benefit from changing treatment from tacrolimus to cyclosporine [1, 40], despite some promising results [46].

It should be noted that no clear relationship exists between tacrolimus drug doses and adverse events and dose titration may be not successful in all patients, necessitating a switch in therapy [38]. 

Although there are many studies trying to address this important issue, no overt guidelines for the management of such a complex metabolic complication are available. The fact that so many influencing factors can have a role in its pathophysiology makes it really difficult to tailor a specific rescue therapy.

We have retrospectively evaluated the incidence of new-onset diabetes after transplantation on a population of 344 kidney transplant recipients. NODAT developed in 29 patients (6.6%), with a mean time of insurgence of 9 ± 4.2 months after transplantation. Male patients with polycystic disease were at higher risk to develop diabetes after transplantation. 

We have applied a conversion protocol to sirolimus in kidney transplant recipients who were on CNI-based immunosuppressive therapy and developed a NODAT.

Interestingly, switch to sirolimus therapy determined a rapid improvement of insulin requirement after conversion compared to patients who remained on lower dose of CNI.

At five-year followup, NODAT resolved in 80% of patients converted to sirolimus, compared with 37.5% of patients on reduced CNI therapy. No patients in both groups experienced an acute rejection, suggesting that both conversion to sirolimus and reduction in CNI dose may be a valid therapeutic choice in the management of NODAT.

It could be questioned that this may reflect an overall higher immunosuppression level in our transplant population. However, the incidence of NODAT in our population was lower than that reported in the literature and NODAT was not related to level of immunosuppression, expressed as trough level of CNI, but more probably is a direct consequence of acute drug toxicity. Moreover, no patient in our cohort died due to infective complications, suggesting that immunosuppressive level was adequate to maintain a good graft function with a low risk of acute rejection.

The absence of acute rejection episodes after conversion to SIR and after dose reduction of CNI may be probably related to the fact that most of NODAT episodes occurred late after transplant, when the risk of acute rejection is lower. 

Conversion to sirolimus resulted in a significant improvement in graft function in NODAT patients compared to patients who remained in CNI reduced dose. 

The molecule of sirolimus, by inhibiting the serine-threonine kinase m-TOR, which plays a key role in the insulin-signalling cascade, has the potential to affect strikingly glucose metabolism [47–49]. In experimental studies, sirolimus in association with tacrolimus induced changes in glucose and insulin responses to glucose challenge that were accompanied by changes in islet apoptosis and insulin content [50] and this effect was reversible after sirolimus discontinuation. Again, rapamycin reduces glucose uptake in human adipocytes through impaired insulin signalling [51]. 

Many recent studies tried to address the possible connection between sirolimus and diabetes after transplantation. Johnston et al. [48] analysing data from >20000 kidney transplant recipients in the US Renal Data System database found that combinations that included sirolimus are associated with higher risk of NODAT compared to combination therapy without sirolimus. As expected, the most diabetogenic combination is with calcineurin inhibitors. However, when the authors stratified the patients including those who did not change therapy during the first posttransplant year (*n* = 16,681), sirolimus was associated with an increased risk of diabetes only in the presence of a calcineurin inhibitor. 

However, large clinical trials did not reveal any increase in the incidence of posttransplantation diabetes among patients who were treated with SIR and a lower risk of NODAT if compared to cyclosporine and tacrolimus [20], and sirolimus could prevent the development of NODAT in kidney transplant recipients [41]. Recent observations, however, reported that the addition of rapamycin to tacrolimus [20] or to cyclosporine [49, 52] could increase the risk of NODAT. 

The rationale of our conversion protocol was developed on two basis: first, in a recent pilot study, we have showed that among 45 kidney transplant recipients treated with *de *novo therapy with sirolimus, no patients developed a NODAT during a mean follow up of 3.4 years [53]; moreover, kidney transplant recipients with type 2 diabetes as a cause of end-stage renal disease were successfully treated with sirolimus without worsening of their metabolic parameters [54]. Finally, a conversion from TAC to cyclosporine, although potentially beneficial in short term [55], is not warranted in the long term, and a low-dose TAC regimen could expose patients to a higher risk of acute rejection [56]. 

In our study, we tried to elucidate some aspects of this multifactorial complication, applying what we believe so an effective rescue treatment. Most of patients with NODAT presented at the onset with a severe hyperglycaemic status, which required an intensive care management. This clinical status suggested acute drug toxicity, similar to that reported in tacrolimus-treated patients with gastrointestinal complications [57], requiring an immediate discontinuation of the CNI with abrupt conversion to SIR. The conversion was not associated with higher incidence of acute graft rejection or major complications. Based on these preliminary results, we decided to apply similar conversion protocol to those patients who developed NODAT with excellent results. 

There are some possible explanations for our impressive results. Sirolimus could counteract the development of NODAT in stable glucose homeostasis due to its positive effects on insulin-stimulated glucose uptake [58]. In a rat model, sirolimus lowered expression and activity of glomerular transforming growth factor-beta 1/2 and vascular endothelial growth factor, all of which are considered central cytokines in the pathogenesis of diabetic nephropathy [59]. Recently, Piemonti et al. [60] evaluated the beta cells function in 22 patients awaiting for islet cells transplantation treated with sirolimus monotherapy, compared with 14 patients not treated. In the group of patients treated with sirolimus monotherapy, the authors observed an increase in fasting C-peptide levels and a significant decrease in exogeneous insulin requirement compared to patients not receiving sirolimus.

To date, there are no studies investigating the incidence of NODAT in kidney transplant recipients treated with *de *novo sirolimus CNI-free immunosuppressive therapy, and there are few data on conversion from CNI-based immunosuppression to sirolimus in patients with NODAT. Teutonico et al. [52] reported a study in 26 kidney transplant cyclosporine-treated recipients and 15 recipients of marginal kidneys who were treated with low dose of TAC and SIR: they all discontinued CNI and were converted to full dose of SIR. The switch to SIR was associated with a 30% increase of incidence of impaired glucose tolerance and four patients developed a NODAT. However, this study was conducted in stable renal transplant recipients without NODAT, and an oral glucose tolerance test was adopted to investigate the glucose metabolism.

Although the results of this study are promising, we are conscious of its limitations: first, the relatively small sample size does not allow drawing definitive conclusions. However, this is a single center analysis and the cohort is homogeneous, reducing the confounding factors related to the type of immunosuppressive protocol used. Moreover, the incidence of NODAT in our cohort was lower than that reported in the literature. This could be related to the early reduction of immunosuppression in our protocol, without increasing the overall risk of acute rejection. 

In conclusion, this study demonstrated that the conversion from CNI to SIR in patients with NODAT could be beneficial, without increasing the risk of acute graft rejection or of other major complications. Randomized trials with larger number of patients are needed to address this important issue. 

## Figures and Tables

**Table 1:** Baseline characteristics of the study population.

	All *n* = 436	NODAT− *n* = 407	NODAT+ *n* = 29	*P* value
Recipient				
Age	48 ± 12	48 ± 6	51 ± 2	0.1
Male sex	277 (63)	255 (61)	22 (76)	0.1
Polycystic kidney disease	85 (19)	75 (18)	8 (27)	0.2
Body mass index	27 ± 6	27 ± 3	28 ± 1	0.2
HCV seropositive	27 (6)	26 (6)	1 (3)	0.5
ABO groups				
A	163 (37)	148 (36)	15 (52)	0.5
B	52 (12)	50 (12)	2 (7)	0.4
AB	13 (3)	12 (3)	1 (3)	0.5
O	208 (48)	197 (48)	11 (38)	0.5
Time on dialysis (months)	51 ± 52	52 ± 53	43 ± 46	0.07
Immunosuppression				
Tacrolimus	304	22 (93)	22 (7)	0.1
Cyclosporine	90	81 (93)	7 (7)	0.4
Sirolimus	42	42 (100)	0	
Mycophenolate mofetil	400	371 (93)	29 (7)	
Steroids	436	407 (94)	29 (6)	
Donor				
ABO group				0.4
Male sex	230 (53)	213 (52)	17 (59)	0.5
Age (>60 years)	153 (35)	145 (36)	8 (28)	0.4
PRA < 20%	417 (96)	388 (95)	29 (100)	0.3
